# Supplementary material for: Functional role of formate dehydrogenase 1 (FDH1) for host and nonhost disease resistance against bacterial pathogens
Source: PLoS One. 2022 May 20;17(5):e0264917. doi: 10.1371/journal.pone.0264917 (PMC9122214; doi:10.1371/journal.pone.0264917)
Supplement: S1 Fig — Sequence information was obtained from the public database; TAIR, NCBI GenBank, and Sol Genomics Network. The software MEGA-X [76] was used for sequence alignment. The amino acid colors were in accordance with the default coloring schemes of ClustalX alignment, which depends on both residue type and the pattern of conservation within a column (http://www.clustal.org/clustal2/). (PPTX) [file pone.0264917.s001.pptx]

## Slide 1
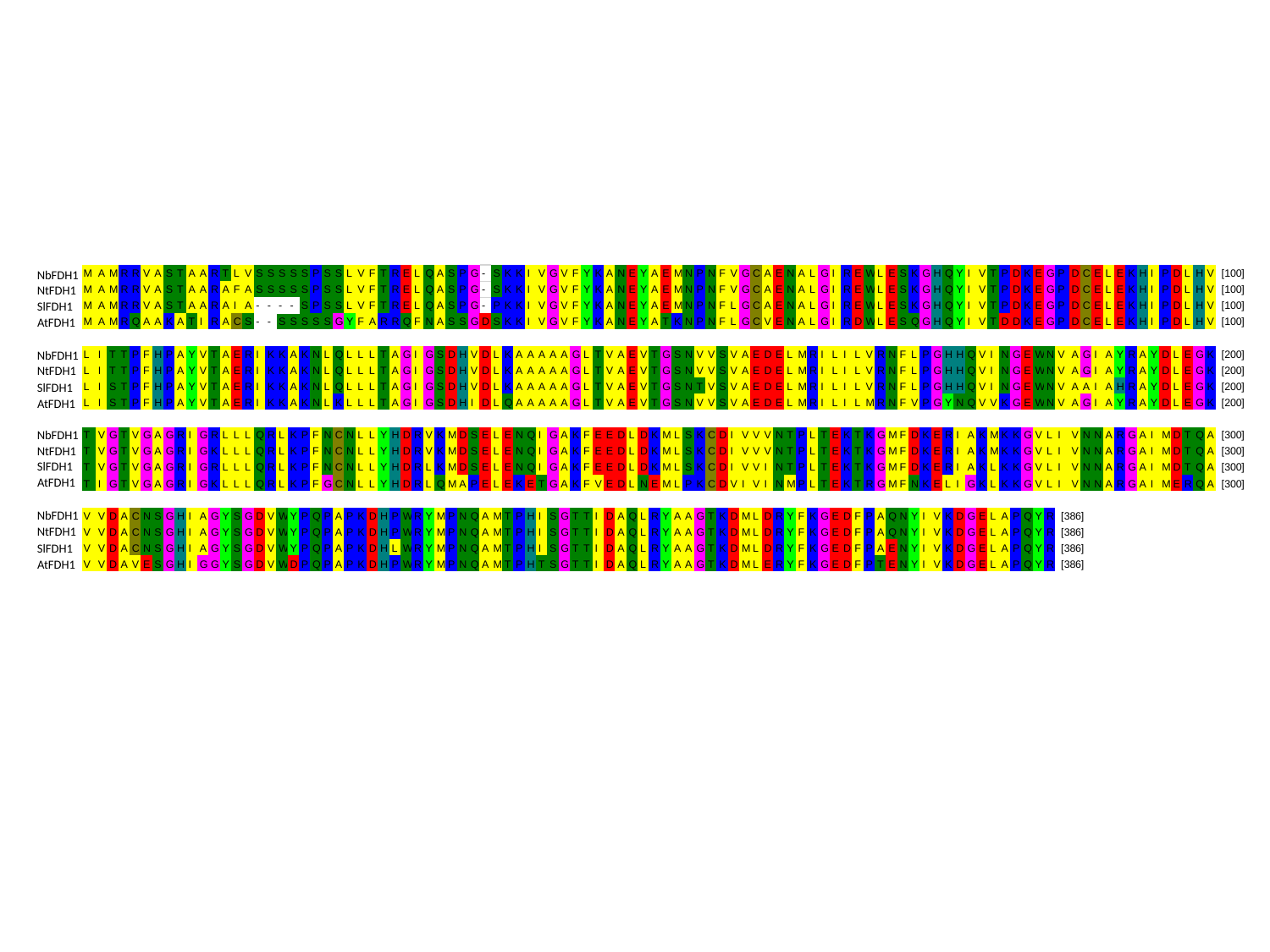

NbFDH1
NtFDH1
SlFDH1
AtFDH1
NbFDH1
NtFDH1
SlFDH1
AtFDH1
NbFDH1
NtFDH1
SlFDH1
AtFDH1
NbFDH1
NtFDH1
SlFDH1
AtFDH1
