# Supplementary figures and images for: Functional role of formate dehydrogenase 1 (FDH1) for host and nonhost disease resistance against bacterial pathogens
Source: PLoS One. 2022 May 20;17(5):e0264917. doi: 10.1371/journal.pone.0264917 (PMC9122214; doi:10.1371/journal.pone.0264917)

## Slide 1
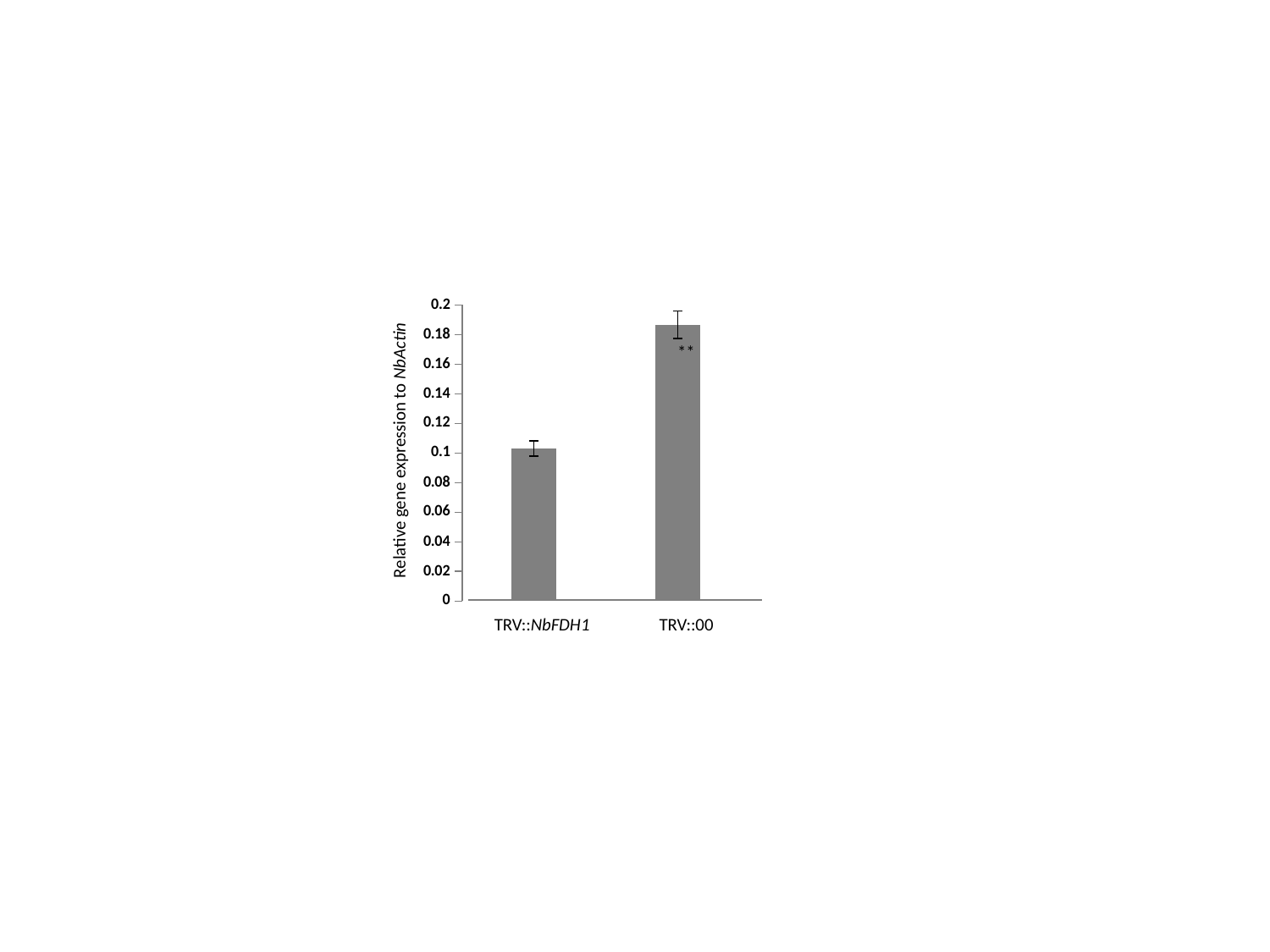

### Chart
| Category | |
|---|---|**
Relative gene expression to NbActin
TRV::00
TRV::NbFDH1

Supplement: S2 Fig — Two weeks old N. benthamiana seedlings were inoculated with TRV1 + TRV::00 (control) or TRV1 + TRV::NbFDH1. Three weeks after TRV inoculation, leaf samples from three different biological replicates for each construct were collected, and gene expression was measured by RT-qPCR. NbActin was used as internal control for normalization. Bars represent mean, and error bars represent standard deviation for three biological replicates (four technical replicates for each biological sample). Asterisk represents statistical significance that was determined using Student’s t-test, (P < 0.01). (PPTX) [file pone.0264917.s002.pptx]

## Slide 1
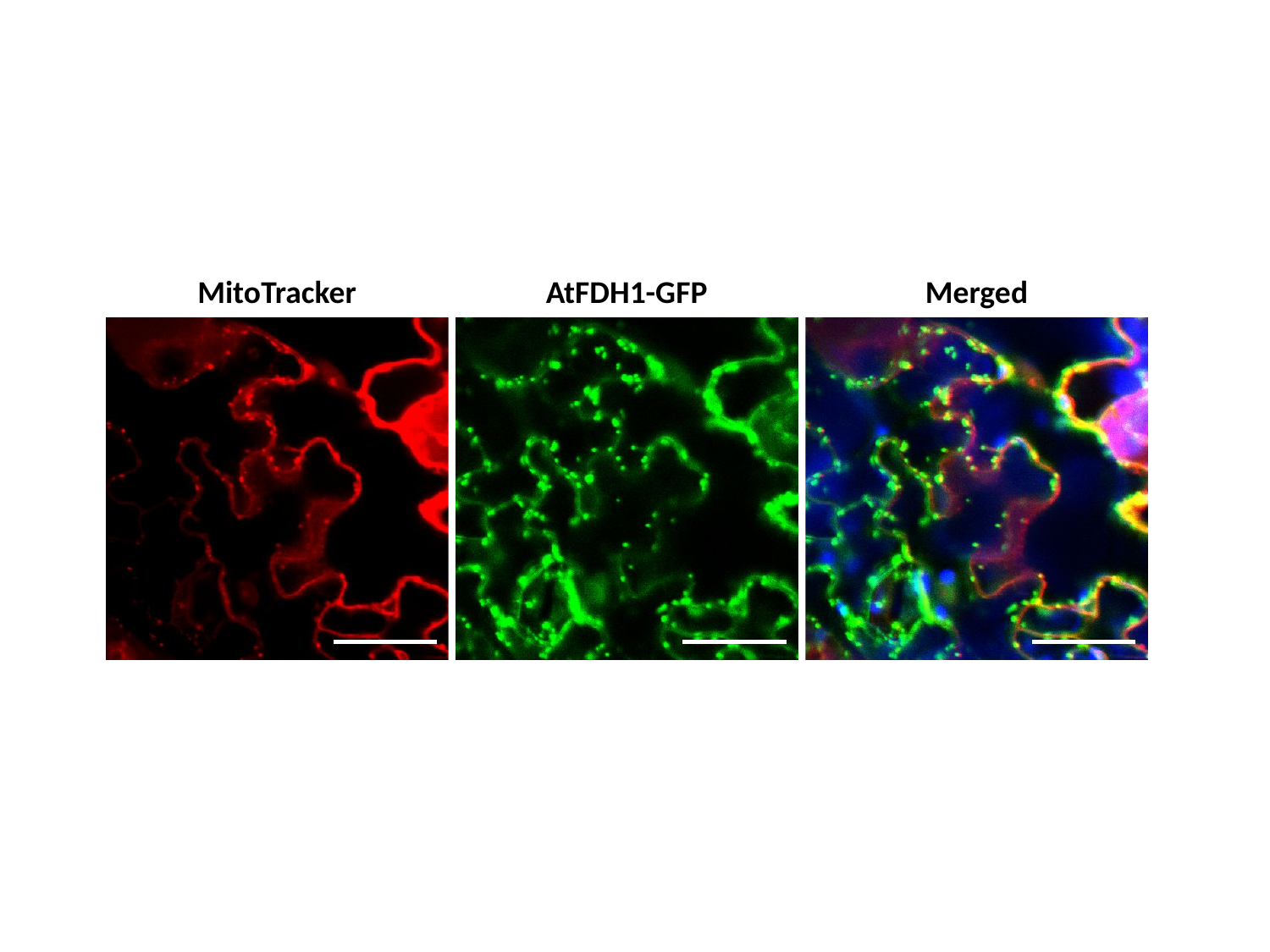

MitoTracker
AtFDH1-GFP
Merged

Supplement: S4 Fig — For Agrobacterium-mediated transient assay, a binary vector containing GFP gene fused to the C-terminal of AtFDH1 was transformed into the A. tumefaciens strain GV3101. The Agrobacterium suspension was (5×10^7^ CFU/ml) was infiltrated using a needleless syringe into N. benthamiana leaves, and the green fluorescence representing AtFDH1 localization was observed 3 days after the agroinfiltration. Red channel (a 561 nm excitation, 570–620 nm emission filter) shows mitochondria stained with MitoTracker dye and green channel shows AtFDH1-GFP localization. Bars = 10 μm. (PPTX) [file pone.0264917.s004.pptx]

## Slide 1
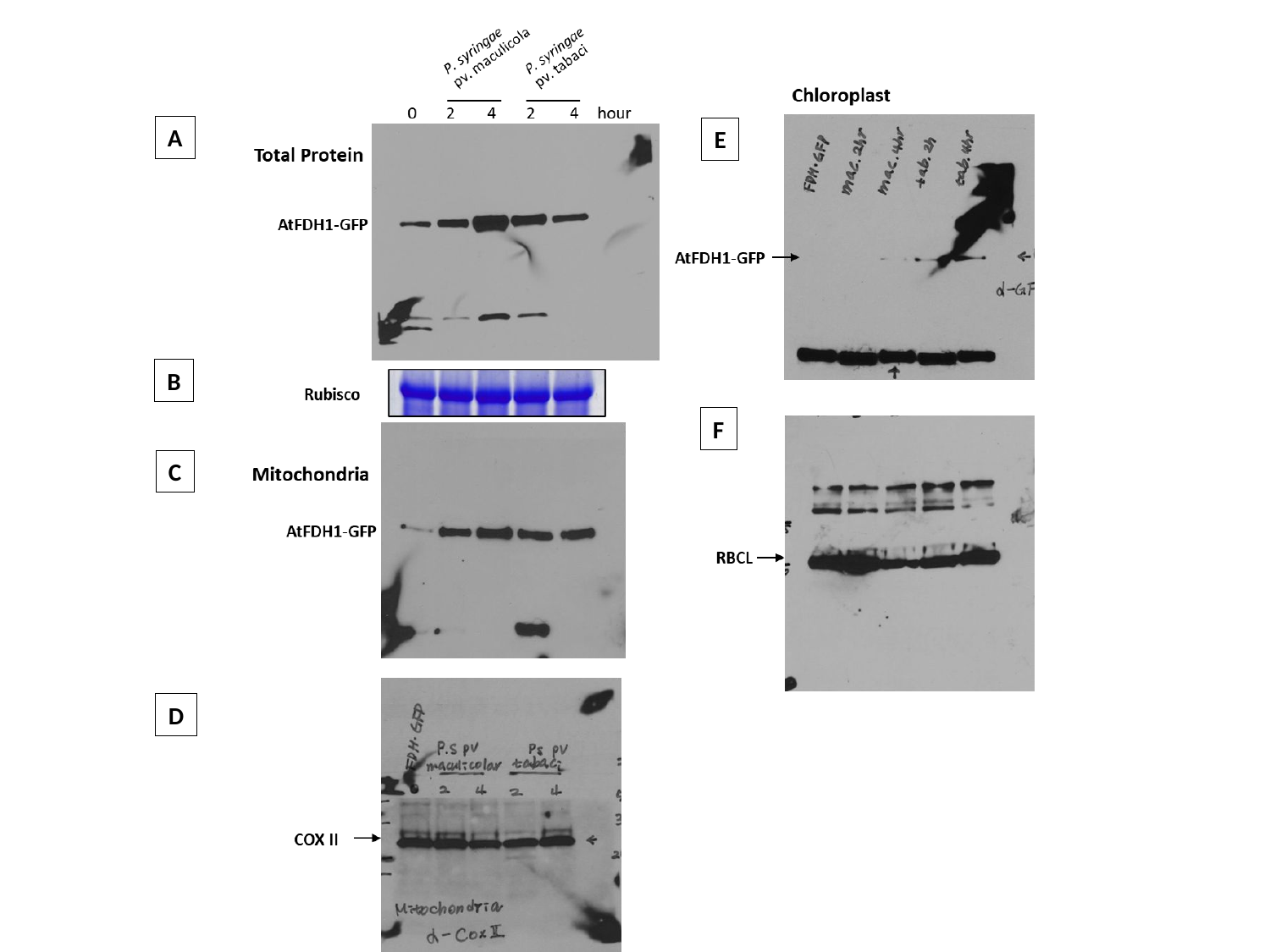

A
E
B
F
C
D

Supplement: S6 Fig — (A) AtFDH1-GFP expression in total protein, (B) Internal control for rubisco expression for total protein, (C) AtFDH1-GFP expression in mitochondrial protein, (D) COXII expression for the internal control of mitochondrial protein, (E) AtFDH1-GFP expression in chloroplast protein, (F) RBCL expression for the internal control of chloroplast. (PPTX) [file pone.0264917.s006.pptx]

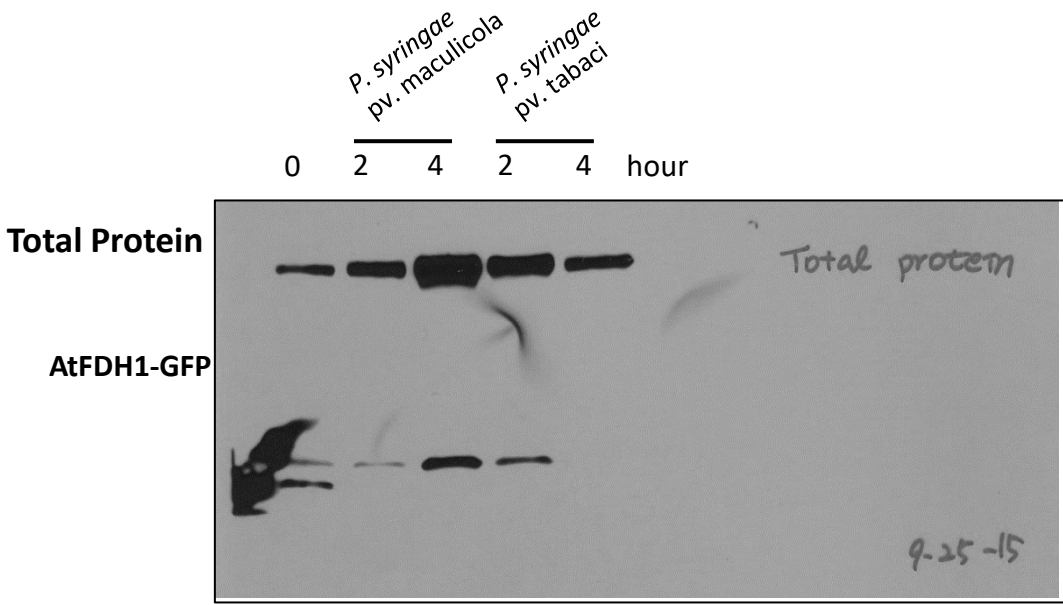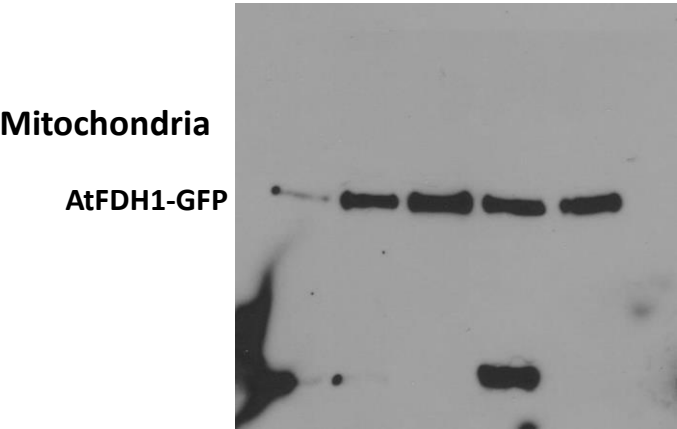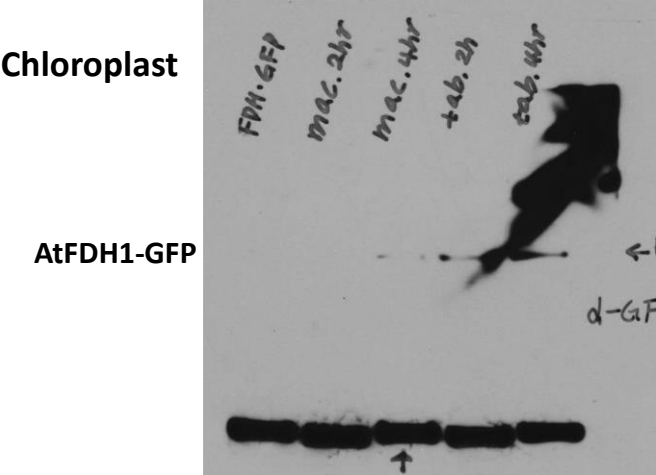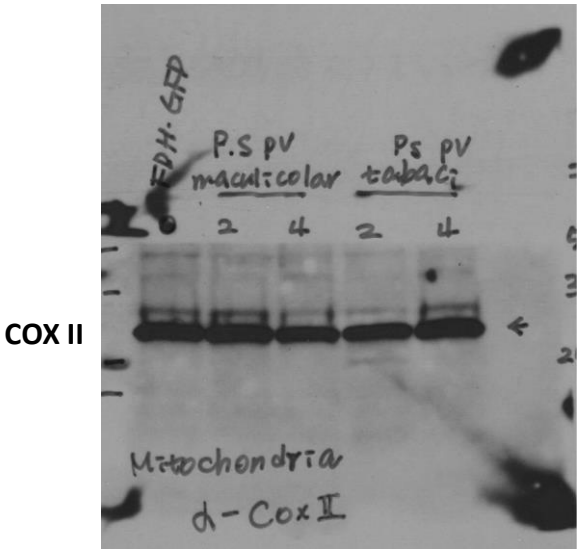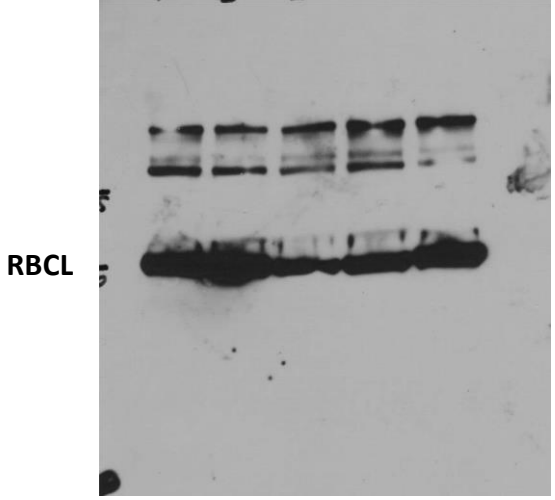

AtFDH1-GFP

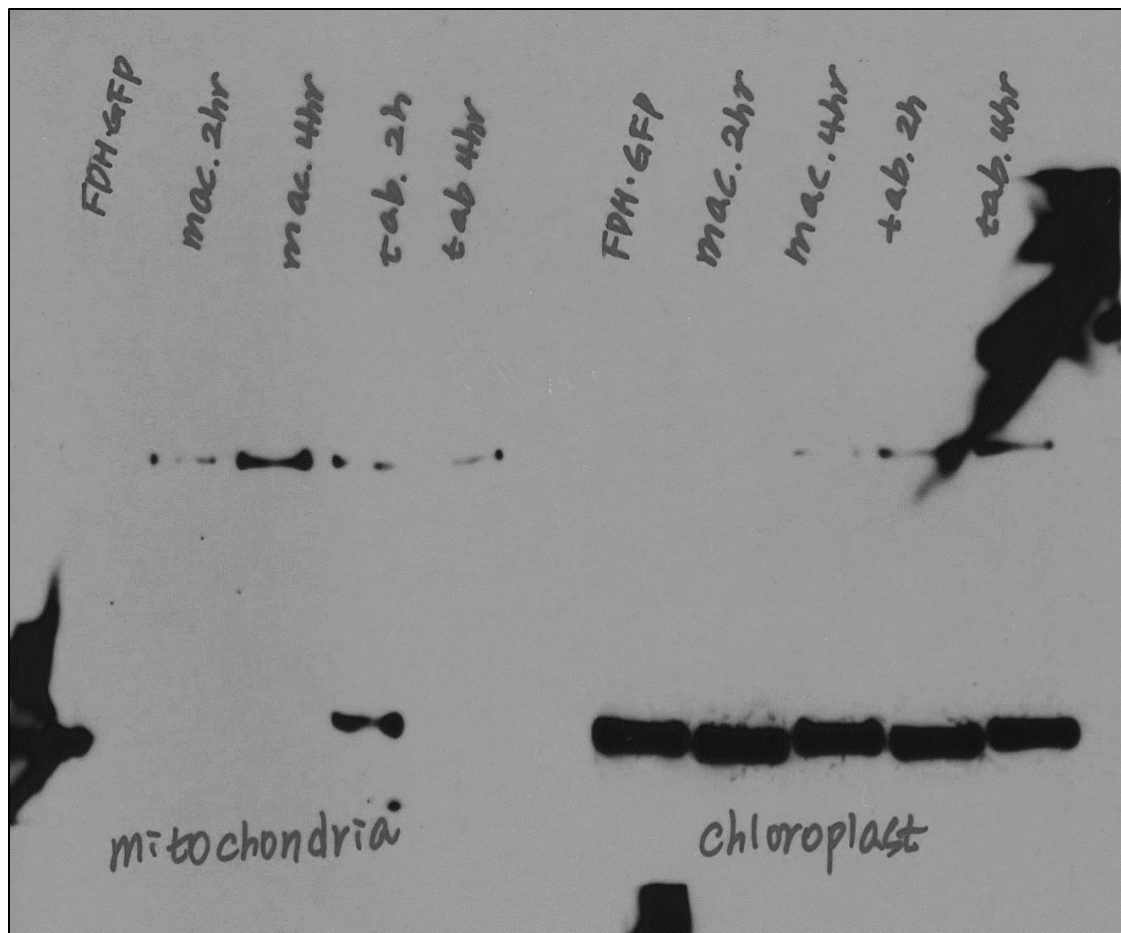

Supplement: S1 Raw images — (PDF) [file pone.0264917.s008.pdf]
