# Supplementary material for: Functional role of formate dehydrogenase 1 (FDH1) for host and nonhost disease resistance against bacterial pathogens
Source: PLoS One. 2022 May 20;17(5):e0264917. doi: 10.1371/journal.pone.0264917 (PMC9122214; doi:10.1371/journal.pone.0264917)
Supplement: S3 Fig — (A) Gene expression patterns of AtFDH1 against P. syringae bacterial pathogen in Arabidopsis. This data was obtained from Arabidopsis eFP Browser at bar.utoronto.ca [77]. (B) AtFDH1 is induced by host and nonhost pathogen inoculations. Four-weeks-old Arabidopsis wild-type (Col-0) were flood-inoculated with host (P. syringae pv. maculicola, Psm) or nonhost (P. syringae pv. tabaci, Pstab) pathogens. The 24 hours after inoculation, leaves were harvested, total RNA was extracted, and subject to RT-qPCR using AtFDH1 specific primers. AtActin was used as an internal control for normalization. Bars represent mean, and error bars represent standard deviation for three biological replicates (four technical replications for each biological replicate). Asterisks represent statistical significance as determined using Student’s t-test, (P < 0.01). (PPTX) [file pone.0264917.s003.pptx]

## Slide 1
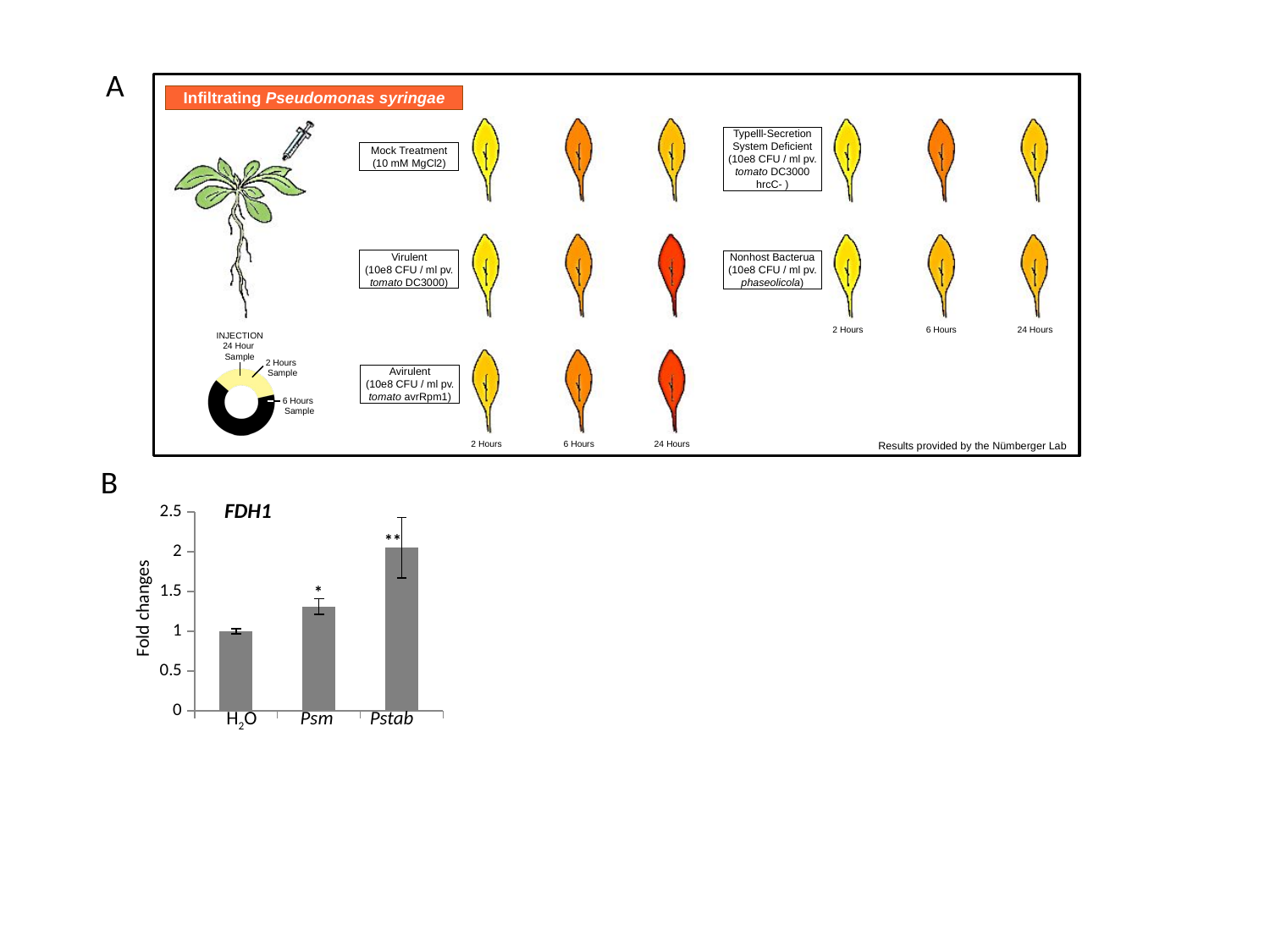

A
Infiltrating Pseudomonas syringae
Mock Treatment
(10 mM MgCl2)
Virulent
(10e8 CFU / ml pv. tomato DC3000)
Avirulent
(10e8 CFU / ml pv. tomato avrRpm1)
2 Hours
6 Hours
24 Hours
Typelll-Secretion
System Deficient
(10e8 CFU / ml pv. tomato DC3000 hrcC- )
Nonhost Bacterua (10e8 CFU / ml pv. phaseolicola)
2 Hours
6 Hours
24 Hours
INJECTION
24 Hour
Sample
2 Hours
Sample
6 Hours
Sample
Results provided by the Nümberger Lab
B
FDH1
### Chart
| Category | |
|---|---|
| Water | 1.0 |
| Psm | 1.3104316884660172 |
| Pstab | 2.050580517313675 |**
*
Fold changes
H2O
Psm
Pstab
