# Supplementary material for: Functional role of formate dehydrogenase 1 (FDH1) for host and nonhost disease resistance against bacterial pathogens
Source: PLoS One. 2022 May 20;17(5):e0264917. doi: 10.1371/journal.pone.0264917 (PMC9122214; doi:10.1371/journal.pone.0264917)
Supplement: S5 Fig — The expression and localization of AtFDH1-GFP was observed in detached (no stress) and peeled adaxial epidermal cells (pathogen stress) from leaves of transgenic Arabidopsis lines expressing AtFDH1-GFP in Col-0. The protein localization was also examined in detached leaf samples 1-hr after the treatment of P. syringae pv. tomato DC3000 (1×10^5^ CFU/ml) and P. syringae pv. phaseolicola (1×10^5^ CFU/ml). Red channel (a 561 nm excitation, 570–620 nm emission filter), showing chloroplast; green channel showing AtFDH1-GFP. Bars = 10 μm. (PPTX) [file pone.0264917.s005.pptx]

## Slide 1
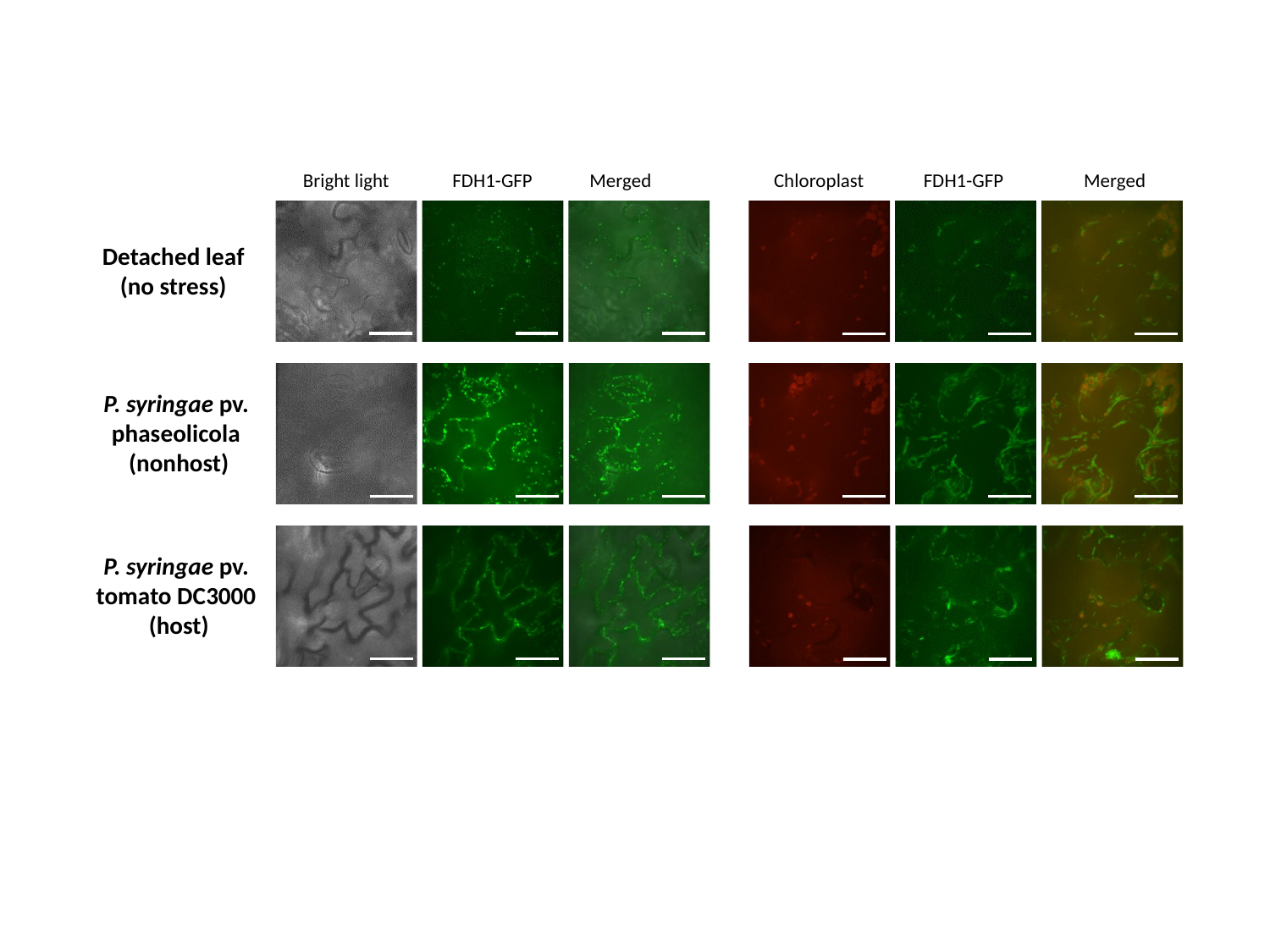

Bright light
FDH1-GFP
Merged
Chloroplast
FDH1-GFP
Merged
Detached leaf
(no stress)
P. syringae pv.
phaseolicola
(nonhost)
P. syringae pv.
tomato DC3000
(host)
