# Supplementary material for: Functional role of formate dehydrogenase 1 (FDH1) for host and nonhost disease resistance against bacterial pathogens
Source: PLoS One. 2022 May 20;17(5):e0264917. doi: 10.1371/journal.pone.0264917 (PMC9122214; doi:10.1371/journal.pone.0264917)
Supplement: Attachment — Submitted filename: Response to Reviewers.docx [file pone.0264917.s012.docx]

Dear Editor,

Thank you for the valuable comments on our manuscript. We have followed the reviewers’ comments and thoughtfully revised the manuscript.

Please see below our responses and let us know for any questions.

Best Regards,

Seonghee Lee

-----------------------------------------------------------------------------------------------------------------------------------------

Dear Dr. Lee,

Thank you for submitting your manuscript to PLOS ONE. After careful consideration, we feel that it has merit but does not fully meet PLOS ONE’s publication criteria as it currently stands. Therefore, we invite you to submit a revised version of the manuscript that addresses the points raised during the review process.

This was an interesting topic that was generally well-received by the reviewers. However, in addition to several minor concerns that both reviewers raised, and that can likely be easily addressed, Reviewer 2 indicated that the localization studies were not yet compelling. I am in agreement that the use of a co-reporter will be useful here. If a suitable reporter is unavailable, other means of improving understanding of FDH1 localization would also be acceptable. In addition, comments made regarding the western blot analysis that should be addressed.

Please submit your revised manuscript by Apr 02 2021 11:59PM. If you will need more time than this to complete your revisions, please reply to this message or contact the journal office at [plosone@plos.org](mailto:plosone@plos.org). When you're ready to submit your revision, log on to [https://www.editorialmanager.com/pone/](https://urldefense.proofpoint.com/v2/url?u=https-3A__www.editorialmanager.com_pone_&d=DwQGaQ&c=sJ6xIWYx-zLMB3EPkvcnVg&r=C68oWkKtABommA8h9xT-wSS3TGJ32YwzrbLMQbtVsc8&m=CjSvh3Ud_adtJub2UpEPBmZ7wwJchse2p5wXYWi1rsA&s=WocAcvNQe9AwkWioAeiGbUlgvya41IhyYSNSk2oTq0w&e=) and select the 'Submissions Needing Revision' folder to locate your manuscript file.

Please include the following items when submitting your revised manuscript:

1. A rebuttal letter that responds to each point raised by the academic editor and reviewer(s). You should upload this letter as a separate file labeled 'Response to Reviewers'.
2. A marked-up copy of your manuscript that highlights changes made to the original version. You should upload this as a separate file labeled 'Revised Manuscript with Track Changes'.
3. An unmarked version of your revised paper without tracked changes. You should upload this as a separate file labeled 'Manuscript'.

Reviewer #1: The manuscript is overall well described the goals, importance and results; properly designed and soundly carried out, emphasizing the important of FDH1 in plant defense responses.

*Minor points are;
In abstract, authors argued that SA signaling is involved in FDH1-mediated defense response. However, Fig. 3 and a corresponding chapter showed the role of both SA and JA in FDH1 activity. Authors should revise the abstract according to the Fig. 3 results, and may discuss potential implications on SA and JA coordination (?) or common understanding of SA/JA antagonisms (if possible, additional JA-marker genes could be added, and/or timely compared with SA-marker genes).*

-> That is correct. We found the important role of both SA and JA for host and nonhost resistance against bacterial pathogens. The statement was corrected.

*Line 245 and 398; the efficacy of NbFDH1-silencing was differently described between line 245 (70%) and line 398 (50%). Those ~20% may have somewhat critical effects on physiology, e.g., may explain different results observed between tomato-silencing and Arabidopsis-KO lines on the infection of host pathogens (Fig. 1C vs. 2B).*

-> As shown in the supplementary Figure 2, the silencing of NbFDH1 is approximately 70%. We corrected the statement in line 402.

*Line 292: chapter “AtFDH1 is … pathogens” compared the upregulation of FDH1 expressions in response to host or nonhost bacteria, and proposed a greater role of FDH1 in nonhost disease resistance. Is it possible that induction/response of FDH1 upregulation by nonhost bacteria is slower (or faster) than by host pathogens? RT-PCR on additional time points could confirm/strengthen the authors’ conclusion.*

-> As shown in supplementary Figure 3 and 5, we found that expression level of FDH1 was much higher in response to nonhost pathogen than by host pathogen. In qRT-PCR assay, we only determined the FDH1 expression at 24hpi; thus, it would be difficult to explain if the gene expression would be faster or slower in response host vs. nonhost pathogens. However, in Figure 6, FDH1 accumulation was elevated faster in response to nonhost pathogen (Pst, 2hpi) at chloroplast sample. The statement was revised in line 370.

*Reviewer #2: The manuscript entitled “Formate Dehydrogenase (FDH1) Localizes to Both Mitochondria and Chloroplast to Play a Role in Host and Nonhost Disease Resistance” by Lee et al. described isolation of a FDH1 gene encoding a formate dehydrogenase from two plant species, Nicotiana benthamiana and Arabidopsis thaliana. The authors concluded that the gene provides nonhost, basal and effector triggered immunity. Furthermore, they concluded that FDH1 in Arabidopsis is localized to mitochondria and following bacterial infection it moves to chloroplast.

It is an interesting study reporting new knowledge in the area of plant immunity.

Major Comments:
1. Localization section requires additional studies. It is hard to follow the photos relevant to mitochondrial and chloroplastic localization of FDH1. It will be best to co-localize FDH1 with a reporter protein; i. e, an RFP fused protein that has been already established to be a mitochondrial or chloroplast protein. Close-up photos will also help the readers to follow the changes in accumulation patterns following infection.*

-> We have used MitoTracker red dye to determine the subcellular localization of FDH1, because previous studies suggested that FDH1 localized in mitochondria and possibly cloroplast. MitoTracker was also used as mitochondrial staining dye and validated the localization of FDH1 in pepper (Choi et al., 2014, Plant Physiology). In addition, there are a number of studies published using MitoTracker as the dye of mitochondria straining (few examples - labeling mitochondria with MitoTracker dyes, Cold Spring Harbor Protocols, 2010; Sorvina et al., 2018, “Mitochondrial imaging…. “, Scientific reports; Jeena et al., 2017, “Mitochondrial localization….”, Nature communication). We agree our localization image was not clearly zoomed in, so another high quality of close-up image (Figure 4) was included to show the localization of FDH1 in mitochondria.

Also, in Figure 5, we did not use RFP fused protein for examining the chloroplast localization. Instead of using RFP fusing protein, we confirmed chloroplast with red autofluorescence of chlorophyll in mesophyll cells. Because it is well known that chloroplasts exhibit very strong autofluorescence in red, with a peak of approximately 680 nm. Thus, we used red channel to show chlorophyll auto fluorescence in chloroplasts of the epidermal cell region (this information was added in line 516). There are numerous studies to use the red autofluorescence of chlorophyll to determine chloroplast. We agree that the image resolution of Figure 5 was not in good quality to understand colocalization of FDH1 in mitochondria and chloroplast. This Figure has been revised for better explanation of the distributional change of FDH1-GFP in response to bacterial pathogens in mitochondria and chloroplast. Please see the revised Figure 4.

*2. The western-blot looks good; but quantification has not made to claim that there is an enhanced accumulation of the fusion protein with time following infection (Figure 6).*

-> That is correct, so we measured western blot band intensities. Figure 6 was revised.

This is a very interesting preliminary observation that FDH1 starts to accumulate in chloroplast following infection. Is not it possible that the protein is directly targeted to chloroplast from cytoplasm following infection? Note that in lane 3, the total protein is much more than that in mitochondria suggesting an increased synthesis of FDH1 following infection.

-> There would be a possibility that FDH1 could be targeted to chloroplast from cytoplasm. Based on our confocal microscope observation in many different experiments, FDH1 is mainly localized in mitochondria, and the expression is drastically increased after nonhost bacterial pathogens. We also see the movement of FDH1 expressing mitochondria is much faster and colocalized to chloroplast after nonhost pathogen infection than host pathogen and water control. Figure 6 is updated and showed the increased synthesis of FDH1 in response to nonhost pathogen (also please see the supplementary Figure 5).

Minor Comments:

*1. It is not evident in this study if FDH1 localization to mitochondria or chloroplast is involved in host and nonhost disease resistance; therefore, title may be revised.*

*-> We agrees the comment, and title has been revised.*

2. The basal resistance section is less developed. How was it measured?

-> In this study, we found that FDH1 is upregulated against both host and nonhost pathogens. Also as shown in Figure 2, fdh1 mutant plants were more susceptible to host pathogen, and the bacterial population in infected leaf tissues were much higher than the bacterial population in wide type. Also similar result showed in another group study in pepper (Choi et al., 2014).

3. Lines 60 - 61: “The efficacy of nonhost disease resistance is based on the recognition of pathogen-associated molecular patterns (PAMPs) and/or pathogen effectors.” This statement should be revised as not all nonhost resistance mechanisms are controlled by receptors for PAMPs. A review of the nonhost resistance mechanisms/genes should be included in “Introduction.”

-> This sentence was revised as shown in line 63-70.

4. Line 252: The sentence starting with “In correlation with ….” should be revised.

-> This sentence was correct.

5. Line 293: May delete the first section of the sentence up to the comma.

-> Corrected as shown in line 280.

6. Lines 307-308: The sentence should be deleted. Basal resistance is not even referred in the previous paragraph.

-> corrected (line 317).

7. Lines 324 -325: “AtFDH1 localizes predominantly in mitochondria, but translocates to chloroplasts in response to abiotic and biotic stresses” – No evidence is provided in support of the translocation of the protein from mitochondria to chloroplast.

-> Yes we agree that, and the sentence was changed.

*8. The manuscript would be benefited from editing for English.*

*- > The manuscript has been thoughtfully read and revised for any potential grammatical errors in English.*
