# Supplementary material for: Functional role of formate dehydrogenase 1 (FDH1) for host and nonhost disease resistance against bacterial pathogens
Source: PLoS One. 2022 May 20;17(5):e0264917. doi: 10.1371/journal.pone.0264917 (PMC9122214; doi:10.1371/journal.pone.0264917)
Supplement: Attachment — Submitted filename: Response to Reviewers.docx [file pone.0264917.s013.docx]

Dear Editor,

Thank you for the valuable comments on our manuscript. We have followed the reviewers’ comments and thoughtfully revised the manuscript.

Please see below our responses and let us know for any questions.

Best Regards,

Seonghee Lee

-----------------------------------------------------------------------------------------------------------------------------------------

Journal Requirements:

Please review your reference list to ensure that it is complete and correct. If you have cited papers that have been retracted, please include the rationale for doing so in the manuscript text, or remove these references and replace them with relevant current references. Any changes to the reference list should be mentioned in the rebuttal letter that accompanies your revised manuscript. If you need to cite a retracted article, indicate the article’s retracted status in the References list and also include a citation and full reference for the retraction notice.

*The list of reference was updated and highlighted for the changes. Following the reviewer’s comments, some old references were excluded because there were new publications available. Also, we found that there were too many references cited in the manuscript, and retracted some references that describe duplicated information.*

[Note: HTML markup is below. Please do not edit.]

Reviewers' comments:

Reviewer #1: (No Response)

Reviewer #2: The revised version is much-improved. I have however a few concerns, which should be addressed.

1. If feasible a video may complement the Figure 5. I could not follow the concept that mitochondria remain attach to the chloroplast for a minute …... see sentence below from the manuscript.

"The arrow in each image (every 1min:30sec) indicates that the mitochondria localized
FDH1 moves to chloroplasts and attach there approximately for 1 min, and later goes apart from the chloroplast."

This sentence was changed and also we included a video (supplementary figure) to describe the movement of mitochondria to chloroplast.

2. Figure 6B is an improvement but it lacks statistical analyses.

Corrected.

3. The manuscript does not have any references to any of the publications of 2020 and 2021. There has been a lot known in the last two years. This can easily be fixed. Certain places authors may consider to cite original references rather than reviews, which are old and do not accommodate new work. Instead of using multiple reviews, authors are encouraged to cite the original work so that readers will be able to access those easily.

Reference list was updated.
